# Plantagora: Modeling Whole Genome Sequencing and Assembly of Plant Genomes

**DOI:** 10.1371/journal.pone.0028436

**Published:** 2011-12-12

**Authors:** Roger Barthelson, Adam J. McFarlin, Steven D. Rounsley, Sarah Young

**Affiliations:** 1 iPlant Collaborative, The BIO5 Institute, University of Arizona, Tucson, Arizona, United States of America; 2 Apple Inc., Cupertino, California, United States of America; 3 The BIO5 Institute and School of Plant Sciences, University of Arizona, Tucson, Arizona, United States of America; 4 Broad Institute, Cambridge, Massachusetts, United States of America; UCLA-DOE Institute for Genomics and Proteomics, United States of America

## Abstract

**Background:**

Genomics studies are being revolutionized by the next generation sequencing technologies, which have made whole genome sequencing much more accessible to the average researcher. Whole genome sequencing with the new technologies is a developing art that, despite the large volumes of data that can be produced, may still fail to provide a clear and thorough map of a genome. The Plantagora project was conceived to address specifically the gap between having the technical tools for genome sequencing and knowing precisely the best way to use them.

**Methodology/Principal Findings:**

For Plantagora, a platform was created for generating simulated reads from several different plant genomes of different sizes. The resulting read files mimicked either 454 or Illumina reads, with varying paired end spacing. Thousands of datasets of reads were created, most derived from our primary model genome, rice chromosome one. All reads were assembled with different software assemblers, including Newbler, Abyss, and SOAPdenovo, and the resulting assemblies were evaluated by an extensive battery of metrics chosen for these studies. The metrics included both statistics of the assembly sequences and fidelity-related measures derived by alignment of the assemblies to the original genome source for the reads. The results were presented in a website, which includes a data graphing tool, all created to help the user compare rapidly the feasibility and effectiveness of different sequencing and assembly strategies prior to testing an approach in the lab. Some of our own conclusions regarding the different strategies were also recorded on the website.

**Conclusions/Significance:**

Plantagora provides a substantial body of information for comparing different approaches to sequencing a plant genome, and some conclusions regarding some of the specific approaches. Plantagora also provides a platform of metrics and tools for studying the process of sequencing and assembly further.

## Introduction

Since the completion of the mapping of the human genome in the 1990's [1], [2], genomics has rapidly matured, and with it, so has the technology used for providing the fundamental sequences for studying genomics: whole genome sequencing. The sequencing and annotation of the Arabidopsis genome [3], [4] was completed in 2000, and provided an improved genetic landscape for studying all plants. Sequencing of whole genomes has proven to be a critical tool for solving biological problems on a large scale, and the sequencing of a number of genomes for model organisms, bacterial genomes, and the genomes of a growing list of crop plant genomes has been realized. As the genomic research has matured, so has sequencing technology. The new generation of technologies has faster sequencing capability, but limitations in read length. The first new technology to be adopted, the 454 sequencing platform [5], was first considered unproven technology, but now has been applied successfully to the sequencing and de novo assembly of many new genomes, viral, bacterial, and larger. Because of its high volume of output and relatively low cost, the Illumina platform is used widely, too, despite its even shorter read length [6]. Similarly the SOLID system has been growing in use [7], and other sequencing technologies from Pacific Biosciences, Helicos, Ion Torrent, and others have been gaining acceptance.

As the sequencing and assembly of a whole genome becomes technically more approachable, and the cost more accessible, more genomes are being sequenced and new applications for sequencing projects are being found. Genome sequencing projects entail challenges that apply to the genomes of all species of plants, animals, and microorganisms. Genome size, genome duplication, and repeat content are all factors to be considered for all genomes that are being appraised for sequencing [8], [9].

A de novo genome sequencing and assembly project for plants has special challenges, because of the relatively high percentage of repeats and the duplication of large portions of some plant genomes [8], [10]. For example, almost 85% of the maize genome originated from transposons [11]. Portions of the wheat genome are comprised of as much as 92% repeats [12]. Transposons and other repeat sequences make it more difficult to assemble reads into contigs for some sections of the genomes, because the assembly software is forced to sort through reads which overlap at high identity, but actually come from different portions of the genome. Many plants also have polyploid or closely matching alloploid duplications of large sections of chromosomes, which can result in contig breaks at polymorphic regions, or misassemblies between large scale duplications. Additionally, plant genomes have expanded families for certain types of genes, such as the protein kinases, the cytochromes P450 [13], and the enzymes engaged in the synthesis of plant secondary metabolites. These and other classes of proteins may have high levels of homology that contribute to unique problems in assembling plant genomes.

With the availability and lower cost of the new sequencing technologies, more laboratories will be using whole genome sequencing as an important part of their research projects, but the challenges provided by all genomes, especially plant genomes can still limit severely the productivity of these studies. There are also many choices to be made in initiating genome sequencing, such as what technology to use, what read length, overall genome coverage, what paired-end library sizes to use, and what assembly program to use. The choices made at the beginning of the study can determine the degree of success of the completed projected.

The Plantagora project strives to provide a stronger basis for designing a genome sequencing strategy by building a database of sequencing studies to use as a guide for the required decision-making. To accomplish this, we created a pipeline for generating short sequences that mimic the reads obtained through two commonly used next gen sequencing platforms, the 454/Roche and Illumina systems. The simulated reads were derived from actual plant genome sequences that have a range of genome sizes. Further, we used the simulated reads in read assembly tests with the appropriate assembly programs to construct contigs and scaffolds. During the assembly process, information was recorded to help evaluate the efficiency of the assembly process. After the assembly process, the resulting contigs and scaffolds were analyzed to produce a long list of metrics, to aid in the comprehensive evaluation of the sequencing approach that was simulated.

All of the results of the Plantagora studies have been entered in summary and in the form of raw data at a newly created website, http://www.plantagora.org. Plantagora is constructed specifically to aid researchers in the critical decision-making required for planning a plant genome-sequencing project. The website provides both information and tools designed for use by genome biologists to view the results of our simulated sequencing studies, and to perform their own simulated genome assembly studies.

## Results

We produced a series of 454 and Illumina sequencing datasets consisting of simulated reads that incorporated the characteristics of the data type, including error rates, insert sizes and read lengths. These simulated reads were derived from actual plant genomic sequences. The 454 platform datasets modeled 500 bp fragment reads and paired-end reads with 2 kb, 8 kb, 20 kb, and 40 kb inserts. The Illumina sequences modeled 50, 75, and 100 bp reads, and all datasets were produced in pairs with same insert sizes as for 454 sequences, plus 500 bp inserts. Simulated reads were derived from chromosome one of the rice genome, and whole genomes, including those of *Arabidopsis thaliana*, *Oryza sativa*, and *Sorghum bicolor*.

Two datasets were combined for each assembly into contigs and scaffolds through the use of several different sequence assembly programs and analyzed for a large series of metrics. Most dataset combinations were from a single technology type, however, one set included a mixture of 454 and Illumina data. Different assemblers were used to assemble the datasets according to the design and flexibility of the assembly programs. The 454 sequence datasets were assembled with Newbler, the Illumina data were assembled with ABySS, and SOAPdenovo, and the combined 454 and Illumina data were assembled with ABySS. Each assembler has unique characteristics and limitations that can affect assembly quality. The Velvet assembler was also considered for use with these studies, but its memory needs were too large to be used efficiently with the number of data sets and/or the number of reads to be assembled.

**Figure 1:**
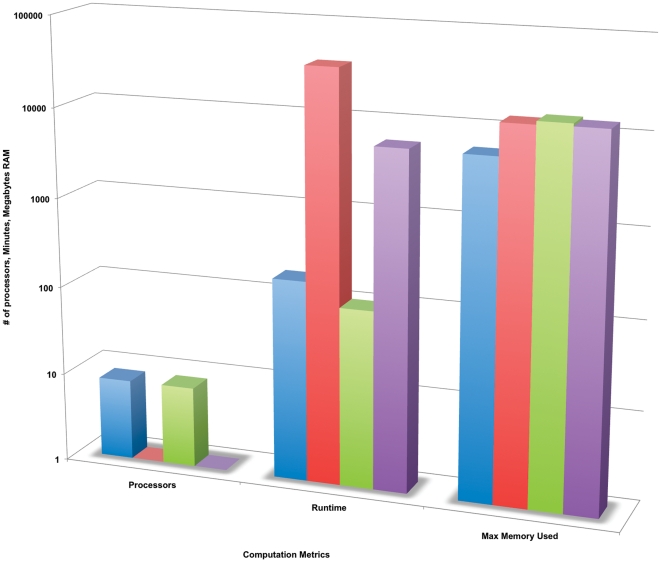
Computation metric values are presented for 4 different assemblies, each from a different sequencing platform/assembler combination, but created from similar datasets. All datasets had a total coverage of 40× for rice chromosome one. Key: **blue** – 500 bp 454 reads, 16× coverage 2000 bp insert spacing with 24× coverage 8000 bp insert spacing, assembled with Newbler; **red** – 16× coverage 500 bp 454 fragment reads with 24× coverage, 75 bp Illumina reads with 8000 bp insert spacing, assembled with ABySS; **green** – 16× coverage 75 bp Illumina reads with 2000 bp insert spacing, 24× coverage 75 bp Illumina reads with 8000 bp insert spacing, assembled with ABySS; **purple** – 16× coverage 75 bp Illumina reads with 2000 bp insert spacing, 24× coverage 75 bp Illumina reads with 8000 bp insert spacing, assembled with Soapdenovo. Metric values were recorded during the assembly process.

All assemblies were monitored as they were produced to record computational metrics, such as run and processor time, maximum memory used, and the final disk space required for storage of the assembly. Figure 1 compares the values of some of the computational metrics recorded for the same or similar datasets from the different platform/assembler combinations used for the Plantagora studies. In general, the Newbler assembler required less memory than did ABySS and SOAPdenovo in our studies, but memory requirements for all the assemblers were similar, and largely determined by genome size and coverage.

**Figure 2:**
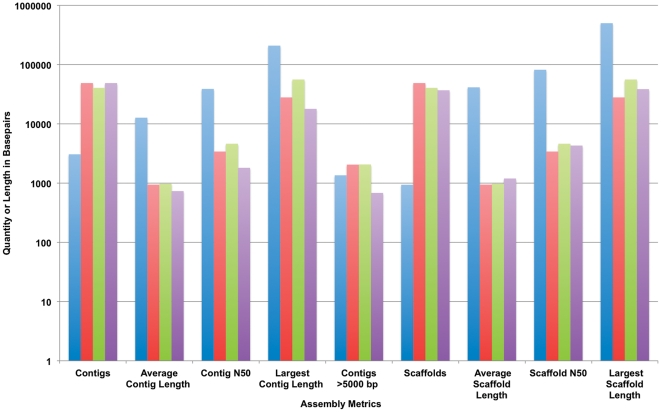
Computation metric values are presented for 4 different assemblies (same assemblies described in **Figure 1** **).** Key: **blue** – 500 bp 454 reads, 16× coverage 2000 bp insert spacing with 24× coverage 8000 bp insert spacing, assembled with Newbler; **red** – 16× coverage 500 bp 454 fragment reads with 24× coverage, 75 bp Illumina reads with 8000 bp insert spacing, assembled with ABySS; **green** – 16× coverage 75 bp Illumina reads with 2000 bp insert spacing, 24× coverage 75 bp Illumina reads with 8000 bp insert spacing, assembled with ABySS; **purple** – 16× coverage 75 bp Illumina reads with 2000 bp insert spacing, 24× coverage 75 bp Illumina reads with 8000 bp insert spacing, assembled with Soapdenovo.

Post assembly, the resulting consensus sequences were analyzed to obtain pertinent assembly quality metrics. Metrics included mean contig and scaffold sizes, length weighted contig and scaffold means, and longest scaffold size. Figure 2 gives a sample of the assembly metrics. In this data sample (same datasets as in Figure 1), Newbler produced fewer contigs and scaffolds that were significantly larger, also.

**Figure 3:**
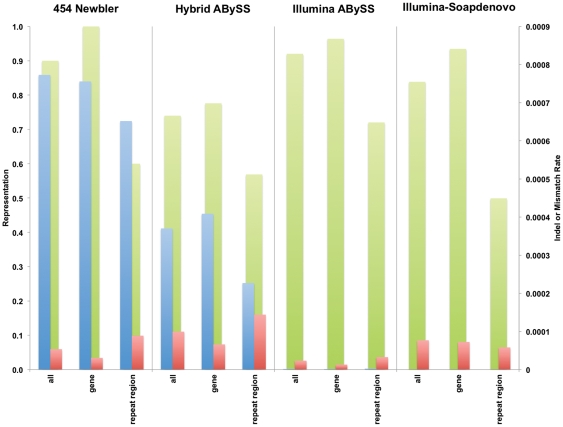
Fidelity metrics were derived by comparing the assemblies against the original genome sequence by alignment. Mean values are presented for representation, indel rate, and mismatch rate for each of the platform/assembler combinations used for the rice chromosome one studies. Key: **green** – mean representation; **blue** – mean indel rate; **red** – mean mismatch rate.

Another set of metrics was produced by aligning the assembly sequences to the reference genome that the raw reads were derived from. This allowed assessment of various types of consensus errors, such as single base errors, indels, and misassemblies. As Figure 3 illustrates, the different assemblers tend to produce more or less of a given type of error, at least with a given genome. Another way to assess the assembly is to calculate the percent of the genome it captures. This representation value is a critical factor in understanding how comprehensive an assembly is. The pipeline that aligns the assemblies to their references, and calculates these statistics, is available at http://www.plantagora.org/tools_downloads. In addition to comparing against a whole reference, this pipeline can also assess the error rates and representation of specific portions of the genome, such as repeat and protein coding regions. For example, Figure 3 shows that the assemblies produced by sequencing platform/assembler combinations that produced the larger contigs and scaffolds, e.g. Newbler with 454 reads, also had higher representation values for all genome sequences. This observation was confirmed for the assemblies of the whole *Oryza sativa* and Arabidopsis genomes, also. (see Figure S1). Similarly, the repeat regions, often a problem for assembly because of their low complexity, almost always had lower representation values than all genome sequences for our studies with rice chromosome one, and for the studies of the complete rice genome, also.

**Figure 4:**
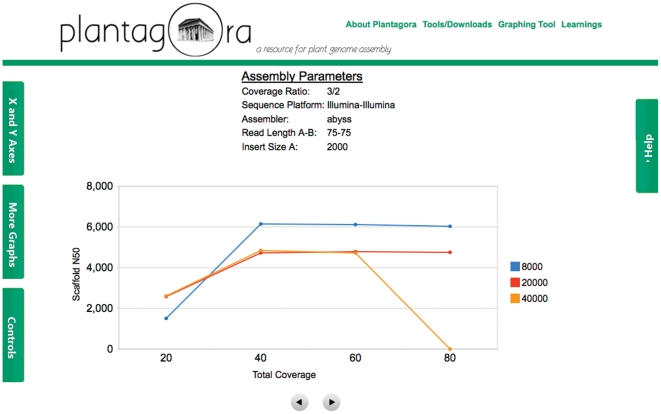
A sample page from the Plantagora website graphing tool is presented. The graphs shown are of the scaffold N50 values vs. total coverage of rice chromosome one for ABySS assemblies of 75 bp Illumina reads, with 2000 bp insert size for dataset A, a 3/2 ratio of dataset A reads to dataset B, and 8000, 20000, and 40000 bp insert sizes for dataset B.

Combined, Plantagora's assembly stats and assembly quality metrics provide a comprehensive means for assessing the assemblies in terms of how complete they are, and how faithful they are to the genome that they should represent. Figure 3 compares 3 of the metrics for the larger portions of the genome analyzed: the whole genome; the gene regions; and the repeat regions.These metrics were captured in the Plantagora assembly database, which is available on the Plantagora website for download (http://www.plantagora.org/tools_downloads). These data are also available for viewing through an interactive graphing tool, also available on the website (http://www.plantagora.org/graphtool.php). An example of the many different graphs that can be produced with the graphing tool is given in Figure 4.

## Discussion

The Plantagora project was established to produce information and guidance aimed at researchers interested in whole genome sequencing projects. Whole genome sequencing using next gen sequencing technologies can provide a large time and cost advantage over more traditional Sanger sequencing of large BAC clones. Unfortunately, the true value of the newer approach is not always easy to realize in actual assembled genome sequences. The shorter reads from next gen sequencing do not always provide the needed, critical contiguity across the genome to allow the formation of large scaffolds.

The main value in Plantagora rests in its ability to provide tangible information about the sequencing and assembly process that can be used by a researcher to guide their approach to sequencing a plant genome, or other types of genomes. To best make use of the information, it needs to be emphasized that these data are a snapshot of current sequencing technologies, de novo assembly software, and costs. Clearly costs will decrease, but the types of choices to be made will remain the same. For example, the importance of assembly size will be balanced against the cost of sequencing, sequencing and computational resources available, and the time the researcher or research team has to devote to the project. Plantagora provides not only information relevant to current technologies, but also the tools to test additional sequencing approaches, with potentially more than two types of data, different sequencing platforms, and new assemblers. As new sequencing technologies become available, they will provide different types of datasets to be assembled. Plantagora's toolset for calculating a wide range of metrics for future test assemblies can be used to assess any consensus sequence and can help guide these improvements of genome assemblies and the sequencing and assembly process.

### Gene and genome coverage metrics

In comparing the effectiveness of different approaches for sequencing and assembly of our model genomes, it is essential to go beyond measuring the size of the assemblies created, and measure the fidelity of the sequence assemblies, including the representation, which measures the amount of the genome that is covered by the assembly, and the error rate, which is made up of the indel rate and the mismatch rate. The metrics from this analysis also include ones related to ambiguities in the assembly, gaps, and negative gaps. All of these measurements were performed on the whole genome assembly, but then broken down into separate genome sections, such as the gene sequences, the repeat regions, and the coding sequences for specific classes of proteins. The protein classes were chosen based on previously identified plant multi-gene families, but a few other large, unique protein classes were also included [13]. These classes had minimally 100 different members in Arabidopsis, but as many as over 1000 for the protein kinase family, for example.

In general, what we learned by the assembly of specific protein families was specific to the genome being studied, and not something that could be easily generalized for a platform. There were few if any truly consistent differences in assembly fidelity metrics for the different protein families. This type of analysis may be more valuable for learning of weaknesses in a specific assembler or a specific assembly, or possibly learning what might be expected in assembling a closely related genome. It does not seem to have a strong value in testing an overall sequencing/assembly approach.

### Assembler Choice

To complicate the sequencing decision-making process, the development of assembly algorithms for next generation sequencing is still in its infancy and there are many assembly programs that represent different approaches to the assembly problem [14]. Limitations of assembly programs, and less than ideal sequencing coverage can reduce the effectiveness of whole genome sequencing studies, even when high coverage of the genome, e.g. on the order of 80×, is available in the raw reads. Examples of these limitations are found in some of the assemblies created for the Plantagora studies documented on the Plantagora website.

One such example was with the SOAPdenovo assembler. The version available for our studies could not use larger k values, which limited its value for read sizes larger than 50. Nor could this assembler (or ABySS) effectively use some of the larger paired end insert sizes that we tested. The assemblies created from Illumina reads with SOAPdenovo had slightly lower representation values compared to assemblies created with ABySS in the studies with rice chromosome one, but the representation values were much lower for the whole rice and Arabidopsis genomes (Figure S1).

Although the ABySS assembler was quite effective with Illumina reads, its performance with combined Illumina and 454 reads produced almost uniformly some of the smallest assemblies with the lowest representation values. Theoretically, the combination of longer and shorter reads could offer the advantages of both longer contiguous sequences from the 454 reads and higher coverage from the Illumina platform's high volume output, but apparently the version of ABySS tested was not effective at combining these advantages.

Newbler, ABySS, and Soapdenovo all use kmers with a de Bruijn-graph-based mapping to form contigs, but neither ABySS nor Soapdenovo uses scaffolding tools that create extended gaps across areas that are poorly resolved [15], [16]. They must be able to form overlapping reads to bridge the distance between paired ends, thereby forming extended contigs. Otherwise they will not benefit from having paired end information. The sizes for these larger contigs depend on coverage and read size, as predicted by Lander and Waterman [17], [18] for assemblies by ABySS. Despite the lower scaffoldN50 values and low average scaffold sizes when compared to Newbler, ABySS produced some scaffolds over 100,000 bp with Illumina reads, and Soapdenovo produced some over 500,000.

### Read Size and Sequencing Platform

Although Newbler and 454 sequencing provide the largest assemblies of next gen reads in our studies, their best results were still confined to specific combinations of reads. At 40× coverage, 454 reads assembled with Newbler readily produced scaffoldN50 values in the millions or 10's of millions of basepairs, if at least one of the datasets had an insert size of 20,000 or 40,000. At lower coverage or with shorter paired-end spacing, the scaffoldN50 values were still typically in the hundreds of thousands, but could be in the tens of thousands. Even at 80× coverage some of the assemblies were under 100,000 bp. Despite large differences in scaffold sizes achieved with different combinations of reads, the 454 platform still generally produced larger scaffolds than the Illumina platform.

### Resolving Repeats

Repeats commonly offer a challenge for genome assembly, but when found in extended blocks within the genome they also become a limiting factor for assembly size. To some extent the barriers they form may be overcome with longer spacing between paired reads, as can be seen with Plantagora's tests with especially long insert sizes, e.g. 20 and 40 thousand bp. The effectiveness of the different sequencing technologies and assemblers to resolve repeats and integrate them into contigs was measured in the Plantagora studies and can be compared to all genome sequences for reference. For example, on average the assemblies of Illumina data with ABySS had the highest representation for repeat regions and the lowest error values for studies with rice chromosome one. The Newbler assembler also produced assemblies with high representation values on average with the repeat regions of the whole rice and Arabidopsis genomes (Figure S1). SOAPdenovo assemblies of the repeat regions of the rice genome had markedly lower representation values compared to all rice genome sequences, but for the Arabidopsis genome there were essentially no differences in representation between all sequences, gene sequences, and the repeat sequences. This was true for the different platforms and assembler combinations tested with Arabidopsis. This different result for Arabidopsis repeats may result from the different character of Arabidopsis repeats compared to those of Oryza species. Arabidopsis has a much lower number of transposable elements than Oryza (http://plantrepeats.plantbiology.msu.edu/composition.html).

The ABySS assembler working with Illumina reads may be slightly more effective than Newbler (with 454 reads) in assembling repeats, but Newbler still has an advantage in assembly size, by bypassing repeats with its long-distance scaffolding tools, and in working with longer contiguous reads. Over time, these differences between the 454 platform and the other platforms will likely change as the nextgen sequencing platforms evolve to produce longer read sequences. The researcher also has a growing number of de novo assemblers to work with and additional, separate scaffolding tools, that could improve the results achieved with the shorter reads currently being used.

### Conclusions

The data produced by Plantagora provides guidance for researchers who are planning a whole genome sequencing and assembly project. The information in the database should help researchers achieve better genome assemblies, based on their own chosen priorities. The primary value of Plantagora exceeds the usefulness of the current data in the databases, and comes from the set of metrics and tools created for the project for running and evaluating the assemblies. The large number of genomes currently being sequenced drives the need for improvements in sequencing methods and the assembly software. An example of efforts to improve the assembly process is found in the recently organized Assemblathon (http://assemblathon.org/), which provides a forum for testing and discussion of different assembly approaches, and should further the development of more effective methods and alternative metrics, as well. The Assemblathon, and future work within the Plantagora platform may help answer the open question of how well does simulated read data model real sequencing data. In the future, with continued efforts, genome sequencing and assembly may become largely automated, but for now, the process still requires careful guidance and decision-making by the genomic researcher.

## Materials and Methods

### Computer Resources

All the assemblies were created by running the datasets with an assembler on one of several servers available for the project. The computer resources used for the project included a server with 4, 6-core AMD 8431 Opteron processors and a total of 256 Gb of memory, a 1,392-core Altix ICE 8200 cluster with 2 Gb of memory per core, a SGI Altix 4700 with 512 cores available and 1,024 Gb of shared memory, and the Pople and Black Light systems on the Teragrid (www.teragrid.org). The Pople system consists of 192 blades, each holding two Itanium2 Montvale 9130 M dual-core processors for a total of 768 cores, and with each blade sharing 8 GB of local memory. Black Light, available for initial testing for this project, consists of 512 Intel Nehalem 2.26 GHz 8-core processors (4,096 cores total) with 8 GB of memory per core (32 TB total).

### Read Simulation

The simulated 454 and Illumina reads were all created in paired datasets, with different paired-end spacing for each dataset. For some of the 454 dataset pairs, the first dataset (A) consisted of fragment data (no paired-ends), and for some of the fragment data, there was no second dataset (B). For the Illumina data, all datasets had paired-ends, and for both A and B datasets the read size was the same. Reads were created using MetaSim_unix_0_9_5 (http://www-ab.informatik.uni-tuebingen.de/software/metasim) from the reference genome sequences for *O. sativa* chromosome one (NC_008394), the *A. thaliana* genome (NC_003070, NC_003071, NC_003074, NC_003075, NC_003076), the complete *O. sativa* genome (NC_008394, NC_008395, NC_008396, NC_008397, NC_008398, v, NC_008399, NC_008400), and the *S. bicolor* genome (NC_012870, NC_012871, NC_012872, NC_012873, NC_012874, NC_012875, NC_012876, NC_012877, NC_012878, NC_012879). The paired-end insert sizes consisted of 2000, 8000, 20000, and 40000 for the 500 bp 454 reads. The same paired-end spacing was used for Illumina reads, except that an additional insert size of 500 bp was included. Illumina reads consisted of 50 bp, 75 bp, and 100 bp. MetaSim was run in conjunction with the shell script make_reads_454.sh or make_reads_illumina.sh. Arguments for these scripts include the chromosome number, the fasta sequence file for the input, and the path to MetaSim. Error models tailored to each platform/read size were used to introduce error at typical rates in the appropriate parts of the reads.

### Sequence Assembly

The assembly of the paired datasets was run by a shell script, assembly_run.sh, with the appropriate assembly software, GS De Novo Assembler 2.3, ABySS 1.1.2, or SOAPdenovo 1.04. The settings for the assemblers included the following: Newbler ‘–m large –e (coverage)’; ABySS ‘mpirun -np 4 abyss-pe -j2 n = 2 k = (40,45, or 50 for read lengths 50, 75, 100 respectively)’; SOAPdenovo ‘max_rd_len = (read length) avg_ins = (insert A size) reverse_seq = 1 asm_flags = 3 pair_num_cutoff = 3 avg_ins = (insert size B) rank = 1 pair_num_cutoff = 3 -K (40,45, or 50 for read lengths 50, 75, 100 respectively)’. The k values were varied in a series of preliminary tests with ABySS, and the results were used to choose the values used routinely as a compromise.

### Evaluation of the Assemblies

During the assembly runs, the resources used, e.g. memory and cpu time, were recorded, and after the assembly process, a perl script was used to calculate statistics on the output contig and scaffold files. All information was entered into a mysql database for each assembly run. Entries included Data Set ID A, Data Set ID B, Assembler, Assembler Parameters, K-value, Processors Used, Runtime, Processor Time, Max Memory Used, Final Disk Space Used, Contigs, Total Contig Length, Average Contig Length, Contig N50, Largest Contig Length, Contigs >1000 bp, Contigs >5000 bp, Scaffolds, Total Scaffold Length, Average Scaffold Length, Scaffold N50, Largest Scaffold Length. The scaffold assemblies were also aligned against the source genome, using nucmer, which is part of the MUMmer package. The whole process was run with a perl script, assess_assembly.pl. Included in the analysis was the ability to record alignment-defined data for portions of the genome identified in a. gff file. The nucmer settings were: nucmer -o -p (base name) (reference genome) (assembly sequences) (gff file) (gff_region). The gff files provided information on subgenomic regions and were obtained as follows: For Arabidopsis the gene information, including the codiing sequences for protein families were from NCBI refseq gff files. The repeat information was from a gff file created by running Repeatmasker. For rice the gene, protein, and repeat annotations were from NCBI genbank files converted to gff files by bp_genbank2gff3.pl. For Sorghum the gene and protein annotations were from JGI gff files, and the gff for repeats was produced by Repeatmasker. The genome regions analyzed were: the whole genome; genes; repeat regions,; and 16 different protein coding sequence families: protein kinase; Zn-finger; bHLH; MADS; ABC transporter; F-box; Cytochrome P450; AP2; MYB; UDP-gluc; TPR; HMG-; phosphatase; RNA binding; Glycoside hydrolase; Leucine-rich repeat.. All data were recorded in the mysql database, including the following metrics: Genome Region; Ambiguous Bases; Error Rate; Indel Rate; Mismatch Rate; Misassembly Rate; Misassembled Contigs; Misassembled Contig Bases; Internal Overlaps; Internal Gaps; Representation; Number of Gaps; Number of Negative Gaps; Average Gap Size; Average Negative Gap Size; Total Gap Bases; Number of Captured Gaps; Average Captured Gap Size; Total Captured Gap Bases; Unaligned Contigs; Unaligned Bases; Ambiguously Aligned Contigs, Ambiguously Aligned Bases. Table S1 provides a brief explanation for each metric.

## Supporting Information

Figure S1 **Representation values for the whole** ***O. sativa*** **genome and the whole** ***A. thaliana*** **genome assemblies were obtained by aligning them against the appropriate genome references.** Representation is the portion of the genome, or in this case genome regions, that is covered by the assembly, and was evaluated with the nucmer aligner from MUMmer. The four different sequencing platform/assembler combinations are compared. Key: **blue** – all genome sequences; **red** – gene regions only; **green** – repeat regions only.
(TIF)

Table S1 **Plantagora studies used a long list of different classes of metrics.** In Table S1, the metrics are divided as they are in the MySQL database. The metrics include: Assembly metrics, or the statistics gathered during the assembly process and the main properties of the assembled sequences; and Accuracy metrics, or metrics derived by comparing the assembled sequences to the reference genome sequences.
(XLS)
