# Supplementary figures and images for: Plantagora: Modeling Whole Genome Sequencing and Assembly of Plant Genomes
Source: PLoS One. 2011 Dec 12;6(12):e28436. doi: 10.1371/journal.pone.0028436 (PMC3236183; doi:10.1371/journal.pone.0028436)

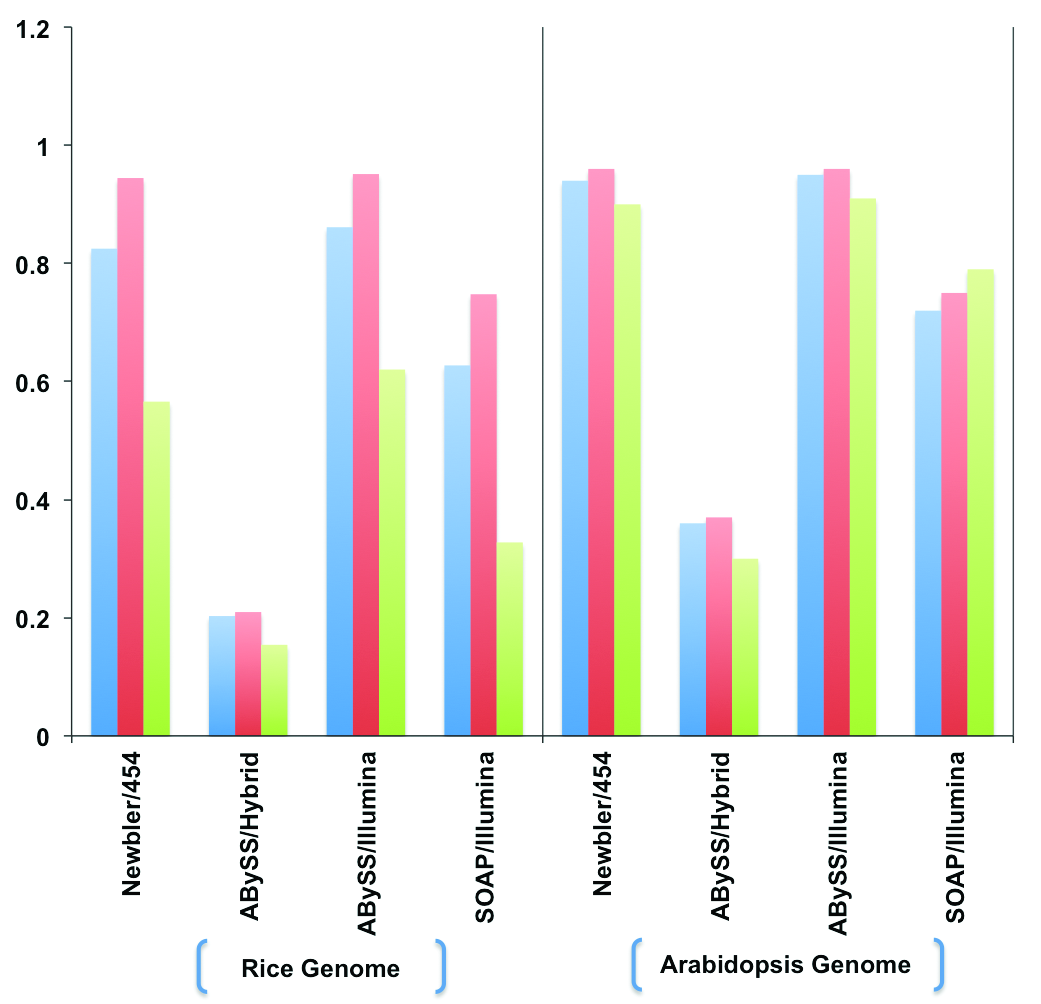

Supplement: Figure S1 — Representation values for the whole O. sativa genome and the whole A. thaliana genome assemblies were obtained by aligning them against the appropriate genome references. Representation is the portion of the genome, or in this case genome regions, that is covered by the assembly, and was evaluated with the nucmer aligner from MUMmer. The four different sequencing platform/assembler combinations are compared. Key: blue – all genome sequences; red – gene regions only; green – repeat regions only. (TIF) [file pone.0028436.s001.tif]
